# Testing Measurement Invariance of the Schizotypal Personality Questionnaire-Brief Scores across Spanish and Swiss Adolescents

**DOI:** 10.1371/journal.pone.0082041

**Published:** 2013-12-12

**Authors:** Javier Ortuño-Sierra, Deborah Badoud, Francesca Knecht, Mercedes Paino, Stephan Eliez, Eduardo Fonseca-Pedrero, Martin Debbané

**Affiliations:** 1 Department of Educational Sciences, University of La Rioja, La Rioja, Spain; 2 Adolescence Clinical Psychology Research Unit, Faculty of Psychology and Educational Sciences, University of Geneva, Geneva, Switzerland; 3 Center for Biomedical Research in the Mental Health Network (CIBERSAM), Madrid, Spain; 4 Department of Psychology, University of Oviedo, Oviedo, Spain; 5 Office Médico-Pédagogique Research Unit, Department of Psychiatry, University of Geneva School of Medicine, Geneva, Switzerland; 6 Research Department of Clinical, Educational and Health Psychology, University College London, London, United Kingdom; Federal University of Rio de Janeiro, Brazil

## Abstract

**Background:**

Schizotypy is a complex construct intimately related to psychosis. Empirical evidence indicates that participants with high scores on schizotypal self-report are at a heightened risk for the later development of psychotic disorders. Schizotypal experiences represent the behavioural expression of liability for psychotic disorders. Previous factorial studies have shown that schizotypy is a multidimensional construct similar to that found in patients with schizophrenia. Specifically, using the Schizotypal Personality Questionnaire-Brief (SPQ-B), the three-dimensional model has been widely replicated. However, there has been no in-depth investigation of whether the dimensional structure underlying the SPQ-B scores is invariant across countries.

**Methods:**

The main goal of this study was to examine the measurement invariance of the SPQ-B scores across Spanish and Swiss adolescents. The final sample was made up of 261 Spanish participants (51.7% men; *M* = 16.04 years) and 241 Swiss participants (52.3% men; *M* = 15.94 years).

**Results:**

The results indicated that Raine et al.'s three-factor model presented adequate goodness-of-fit indices. Moreover, the results supported the measurement invariance (configural and partial strong invariance) of the SPQ-B scores across the two samples. Spanish participants scored higher on Interpersonal dimension than Swiss when latent means were compared.

**Discussion:**

The study of measurement equivalence across countries provides preliminary evidence for the Raine et al.'s three-factor model and of the cross-cultural validity of the SPQ-B scores in adolescent population. Future studies should continue to examine the measurement invariance of the schizotypy and psychosis-risk syndromes across cultures.

## Introduction

The study of clinical and subclinical psychosis phenotypes has been advanced in the last two decades [1]. Schizotypy is a complex construct intimately related to psychosis at a genetic, biochemical, phenotypic, emotional, and behavioural level. [2]–[6]. Schizotypal experiences, such as magical thinking, anhedonia, or paranoid ideation, can be found in the general population, below the clinical threshold, and without necessarily being associated with a mental disorder [7]. Independent follow-up studies show that adolescents and young adults who report schizotypal experiences, compared to those who do not report such experiences, are at greater risk of transition to psychosis and related disorders [8]–[14]. However, it is true that recent studies indicate the low specificity of these experiences and that their evolution is limited not only to the formal diagnosis of psychosis but also to other mental disorders (e.g., depression) [15]. Schizotypy is also a relevant predictive factor on examining adolescents at-high genetic risk [16] and at-high clinical risk for psychosis [17]. Furthermore, healthy adolescents and young adults who report schizotypal experiences also present subtle emotional, behavioural, neurocognitive, and/or social deficits [2], [4], [18]–[23], similar to those found in patients with psychosis and in those with schizotypal personality disorder. In addition, schizotypal traits and experiences share the same risk factor as evidenced in clinical psychosis (e.g., trauma, urbanicity, age) [24]. In this sense, schizotypal experiences and traits would represent the behavioural expression of latent vulnerability to psychosis [1].

The aim of the psychometric high-risk paradigm is the identification of individuals at high risk for schizophrenia-spectrum disorders using their score profile on measurement instruments. At present, it is considered to be a feasible and useful strategy which allows a series of advantages with respect to other assessment methods, as it is a noninvasive method of rapid application and easier administration, scoring and interpretation [2], [10]. Moreover, it allows the study of symptoms that are similar to those found in patients with schizophrenia while avoiding the confounding effects frequently found in these individuals (e.g., medication or stigmatization). It is possible that early detection and intervention of psychosis-risk syndromes can prevent or decrease the probability for transition to psychosis. It is also interesting to study schizotypy at the trait level, because it is associated with positive developments such as creativity. Several self-reports for the assessment of schizotypy have been developed [25], such asthe Wisconsin Schizotypy Scales (WSS) [2], the Oxford-Liverpool Inventory of Feelings and Experiences (O-LIFE) [26] and the Schizotypal Personality Questionnaire (SPQ) [27], or its brief version (SPQ-B) [28]. The SPQ-B has been used with relatives of patients with schizophrenia-spectrum disorders [29], nonclinical adolescents [30], [31], outpatient adolescents [32], and college students [33]–[36]. The SPQ-B's psychometric properties have been examined previously. The internal consistency indices ranged from 0.75 to 0.83 and the test-retest reliability from 0.82 to 0.90. Furthermore, several sources of validity evidence of the SPQ-B scores have been tested (e.g., internal structure, relations to other variables) [25], [28], [31].

Examination of the dimensional structure underlying the SPQ-B scores reveals that schizotypy is a multidimensional construct. Using the SPQ-B, Raine et al.'s [37], three-dimensional model, has been widely replicated, and shows invariance across gender and age [28], [30], [32], [34]–[36]. This model includes the Cognitive-Perceptual, Interpersonal and Disorganization dimensions. Stefanis et al.'s [38], a four-dimensional model, which includes the Cognitive-Perceptual, Interpersonal, Disorganization and Paranoid dimensions, has also been replicated in SPQ-B [30], [34]. For example, Fonseca-Pedrero et al. [30], using the SPQ-B in a sample of non-clinical adolescents, found the three-dimensional and four-dimensional models to be those that best fit the data. Similar results have been found using the SPQ [39]–[45]. However, although the dimensionality of schizotypy has been exhaustively analyzed, it is still unknown whether the dimensions of schizotypy, measured via the SPQ-B, are invariant or equivalent in adolescents originating from different countries.

In this study of measurement invariance or measurement equivalence, one important goal is to analyze whether the measurement instrument and the construct being measured are operating in the same way across samples of interest. When comparisons between groups (e.g., male/female) are made, it is typically assumed that the measurement instrument, the number of factors, the factor loadings, the perceived item content, and the underlying construct behave equally across the groups being compared [46], [47]. Nevertheless, this assumption must be tested. It is crucial to examine the measurement invariance of the assessment tool, so that findings based on comparisons of the groups can be valid. Thus, it would be inappropriate to make comparisons with respect to schizotypal traits if, for example, Swiss and Spanish adolescents interpret the content of the items differently, or if the measurement instrument does not behave in the same way across groups (e.g., different dimensional structures). If measurement invariance across groups does not hold, the validity of the inferences and interpretations drawn from the data may be erroneous or unfounded.

Adolescence is a particularly important developmental stage for socio-emotional development, but it is also marked by the emergence of mental health problems, specifically, psychotic disorders [48]. Likewise, it is an appropriate time for studying possible risk markers for schizophrenia and for the promotion of detection and early intervention strategies previous to the development of the psychosis-risk syndromes (e.g., prodromes) or clinical disorders. For this reason, it is important to have reliable measuring instruments to use in this sector of the population that will allow rapid identification of participants at risk for psychosis -or who present schizotypal traits and experiences- and to gain further insight into the developmental trajectories of schizotypy during adolescence. It is also a priority to conduct studies of measurement equivalence that guarantee the comparability of scores across cultures (e.g., to set cut-off scores, to conduct international research). As yet, there has been no in-depth examination addressing the question of whether the dimensional structure underlying the SPQ-B scores is invariant across countries. The present study examines the cross-cultural equivalence of the factor structure of the SPQ-B across Spanish and Swiss adolescents in order to test the measurement invariance across groups and provide construct validity of the SPQ-B scores. We hypothesized that Raine et al.'s [37] model would provide the best fit to the data in both samples. We further hypothesized that the factor structure underlying the SPQ-B scores would be invariant across samples.

## Method

### Participants

Due to the sample strategy, and in order to guarantee the representativeness of the sample, different cities and different types of school were selected in each country. Participants volunteered to take part in the study (convenient samples). In the Spanish sample, students were from different types of secondary schools – public, grant-assisted private and private – and from vocational/technical schools belonging to the Principality of Asturias and La Rioja. The final sample comprised a total of 261, 135 were male (51.7%), belonging to seven schools. The age of the participants ranged from 14–19 years (*M* = 16.04; *SD* = 1.24). The age distribution of the sample was the following: 14 years (*n* = 24; 9.2%), 15 years (*n* = 73; 28%), 16 years (*n* = 78; 29.9%), 17 years (*n* = 45; 17%), 18 years (*n* = 37; 14.2%), 19 years (*n* = 4; 1.5%). In the Swiss sample, participants were French-speaking community adolescents attending public or private schools in the Cantons of Geneva, Vaud and Jura, Switzerland. To be eligible to participate in the study, youth needed to be aged between 12 and 20, French-native speaking and receive parental consent. The final sample comprised a total of 241, 126 were male (52.3%). The age of the participants ranged from 12–20 (*M* = 15.94; *SD* = 1.94). The age distribution of the sample was the following: 12 years (*n* = 11; 4.6%), 13 years (*n* = 20; 8.3%), 14 years (*n* = 32; 13.3%), 15 years (*n* = 27; 11.2%), 16 years (*n* = 46; 19.1%), 17 years (*n* = 57; 23.7%), 18 years (*n* = 28; 11.6%), 19 *(n* = 17; 7.1%) and 20 (*n* = 3; 1.2%). Neither age (*t* = 0.657; *p* = 0.511) nor sex rates (*χ^2^* = 0.016; *p* = 0.901) differed across subsamples.

### Instrument

The Schizotypal Personality Questionnaire-Brief (SPQ-B) [28] is a 22-item (true/false) self-report based on the SPQ [27] for the assessment of schizotypal personality disorder according to DSM-III-R diagnostic criteria [49]. The SPQ-B consists of three subscales: Cognitive-Perceptual –Positive- (ideas of reference, paranoid ideation, magical thinking and unusual perceptual experiences), Interpersonal (social anxiety, no close friends, blunted affect and paranoid ideation) and Disorganized (odd speech and behaviour). A Spanish version of the SPQ-B previously validated in adolescents was used in this research [30], [50]. The internal consistency for the SPQ-B subscales found in Spanish populations ranges from 0.61 to 0.69, whereas for the total score it ranges from 0.81 to 0.88 [30], [50]. A French version of the SPQ validated in adolescents was used [51]. In the Swiss sample the SPQ version was used, of which those 22 items that made the short version of the SPQ were selected. The internal consistency for the SPQ-B subscales found in Swiss populations ranges from 0.67 to 0.73 and was 0.83 for the total score. Both SPQ-B versions have followed international guidelines for test translation and adaptation [52], [53].

### Ethic statement

In the Spanish sample, written parental/tutor informed consent was obtained for all minors involved in the study. The study was approved by the Research and Ethics Committees at the University of Oviedo and the Department of Education of the Principality of Asturias. In the Swiss sample, written informed consent was received from participants and their parents under protocols approved by the Institutional Review of the Department of Psychiatry of the University of Geneva Medical School.

### Procedure

In the Spanish sample, the questionnaire was administered collectively, in groups of 10 to 35 students, during normal school time in a classroom specifically prepared for this purpose. The completion of the questionnaire was conducted under the supervision of a researcher at all times. The study was presented to participants as part of a research project on the diverse characteristics of personality. The study is part of a wider investigation on the detection and early intervention in psychological disorders in adolescence. In the Swiss sample, participants were administered a battery of self-report questionnaires assessing the expression of schizotypal traits. To ensure that all subjects understood the items, trained clinical psychologists (M.D and D.B) supervised this process. After a phone contact, where research objectives were presented to parents and adolescents, families decided whether they wished to volunteer for the study. Each adolescent received financial compensation for completing the study (15 Euros/hour). This study is integrated in a broader research looking at the link between mentalizing skills and personality traits during adolescence.

### Data analysis

First, we calculated descriptive statistics for the items of the SPQ-B in both samples. Second, with the aim of studying the structure of schizotypy, several confirmatory factorial analyses (CFAs) were conducted at the item level [30]. It should be mentioned that these hypothesized factorial models do not derive specifically from factorial studies carried out with the SPQ-B, but rather with the SPQ (at the level of scales) or with structured interviews. Thus, and given the complexity of the syntax and the small number of items making up the SPQ-B, there are factorial models that cannot be tested (e.g., five-factor model). Third, and with the aim of studying measurement invariance (MI) across groups, successive multi-group CFAs were conducted. Following results of the CFAs, Raine et al.'s [37] three-dimensional model was used. Due to the categorical nature of the data, we used the robust Mean-adjusted Weighted Least Square method (WLSMV) for the estimation of parameters [54]. The following goodness-of-fit indices were used: Chi-square (χ^2^), Confirmatory Factor Index (CFI), Tucker-Lewis Index (TLI), Root Mean Square Error of Approximation (RMSEA) and Weighted Root Mean Square Residual (WRMR). To achieve a good fit of the data to the model, the values of CFI and TLI should be over 0.95 and the RMSEA and WRMR values should be under 0.08 for a reasonable fit and under 0.05 for a good fit [55], [56].

Then with the aim to test MI across subsamples, successive multi group CFAs were conducted [57]. Generally the MI reflects that the construct measured has the same structure and meaning across the groups compared. Basically, a hierarchical set of steps are followed when testing MI across groups, typically starting with the determination of a well-fitting multi-group baseline model and continuing with the establishment of successive equivalence constraints in the model parameters across groups [46], [47], [57]–[59]. The first step established the configural invariance model, in which items were constrained to load on the same factors across groups, but all item thresholds and factor loadings were free to vary across groups. For the models to be identified, we fixed all item scale factors to one and all factor means to zero in both groups. When configural invariance model is found, it is assumed that the general factor structure is at least similar, though not necessarily equivalent, across groups. In a second step, we established a strong invariance model, which contained cross-group equality constraints on all factor loadings and item thresholds, as well as on the covariance between the two factors. As required by the model, scale factors were fixed to one in one group and were free in the other, and factor means were fixed to zero in one group and were free in the other [54]. The assumption of strong invariance model is also necessary for comparing groups on a latent trait (e.g., schizotypy dimensions) [46], [47], [58], [59].

The models analyzed can be seen as nested models to which constraints are progressively added. For the comparison of the nested models, we proposed criteria such as the ΔCFI (practical perspective) or chi-square difference tests (Δχ^2^) (traditional perspective) [58], [60]. As some limitations have been found in the Δχ^2^ regarding its sensitivity to sample size, Cheung and Rensvold [60] proposed a more practical criterion, the ΔCFI, to determine whether the compared models are equivalent. Thus, when there is a change greater than 0.01 in the CFI between two nested models, the least constrained model is accepted and the other is rejected—that is, the most restrictive model does not hold. If the change in CFI is less than 0.01, it is considered that all specified equal constraints are tenable, and we can therefore continue with the next step in the analysis of MI. However, when this criterion is not met and some of the parameters (e.g., factorial loadings or thresholds) are not specified to be equal across groups, partial MI model can be considered [61]. The statistical analyses were carried out using the programs SPSS 15.0 [62] and Mplus 5.2 [54].

## Results

### Descriptive statistics of the SPQ-B items

The mean and standard deviation for the SPQ-B items in both samples are shown in table 1. Internal consistency values for the SPQ-B total and subscales scores in the Spanish sample were 0.67 (Cognitive-Perceptual), 0.74 (Interpersonal), 0.59 (Disorganized), and 0.81 (total score). Internal consistency values for the SPQ-B total and subscales scores in the Swiss sample were 0.74 (Cognitive-Perceptual), 0.76 (Interpersonal), 0.67 (Disorganized), and 0.84 (total score).

**Table 1:** Mean, standard deviation and standardized factor loadings for the confirmatory factor analysis of the three-dimensional model [37] for Spanish and Swiss samples.

	Spain					Switzerland				
Item	*M*	*SD*	F I	F II	F III	*M*	*SD*	F I	F II	F III
1	0.46	0.50		0.71		0.32	0.47		0.69	
2	0.17	0.38	0.77			0.32	0.47	0.64		
3	0.44	0.50			0.71	0.37	0.48			0.70
4	0.25	0.44	0.47			0.36	0.48	0.44		
5	0.21	0.41	0.70			0.26	0.44	0.75		
6	0.20	0.40			0.83	0.12	0.32			0.53
7	0.25	0.43	0.51	0.35		0.27	0.44	0.48	0.26	
8	0.23	0.42			0.71	0.39	0.49			0.67
9	0.27	0.45	0.39	0.40		0.18	0.38	0.72	0.12	
10	0.25	0.44	0.49			0.17	0.37	0.55		
11	0.43	0.50		0.71		0.31	0.46		0.82	
12	0.11	0.31	0.62			0.12	0.33	0.65		
13	0.36	0.48			0.62	0.54	0.50			0.63
14	0.43	0.50	0.25	0.51		0.40	0.49	0.18	0.65	
15	0.41	0.49		0.55		0.17	0.37		0.71	
16	0.35	0.48	0.46			0.29	0.45	0.70		
17	0.22	0.42	0.40	0.17		0.41	0.49	0.45	0.25	
18	0.11	0.32		0.73		0.09	0.29		0.81	
19	0.13	0.34			0.84	0.45	0.50			0.62
20	0.27	0.44			0.77	0.28	0.45			0.71
21	0.36	0.48		0.65		0.29	0.46		0.61	
22	0.52	0.50		0.69		0.57	0.50		0.52	

*p*<0.01) except item17 (Factor II) (Spanish sample), and items 9 (Factor II) and 14 (Factor I) (Swiss sample). Note: All standardized factor loadings were statistically significant (

### Confirmatory factor analysis of the SPQ-B items

The goodness-of-fit indices for the proposed models are presented in table 2. As can be seen, the models which showed the best fit in both samples were Raine et al.'s [37] three-factor model and Stefanis et al.'s [38] four-factor model. The goodness-of-fit indices were better for the Swiss sample. For both models, in the Spanish sample, the CFI value was higher than 0.92 and the RMSEA was 0.06. In the Swiss sample, the CFI value was higher than 0.95 and the RMSEA was 0.03. In the case of Raine at al.'s [37] model, where the items measuring paranoid ideation saturate in both the Cognitive-Perceptual and the Interpersonal dimensions, the correlation between the latent variables ranged from 0.35 (Positive-Interpersonal) to 0.68 (Interpersonal-Disorganization) in the Spanish sample and from 0.51 (Positive-Interpersonal) to 0.77 (Positive-Disorganization) in the Swiss sample. In the four-factor model the correlation between the latent variables ranged from 0.34 (Paranoid-Interpersonal) to 0.86 (Paranoid-Positive) in the Spanish sample, and from 0.50 (Paranoid-Interpersonal) to 0.94 (Paranoid-Positive) in the Swiss sample. Moreover, in the four-factor model, weight factor loadings were lower than those found in the three-factor model in both samples. For instance, the four-factor model in Swiss adolescents showed four completely standardized loadings not statistically significant (*p*≤0.01). In accordance with (a) the parsimony criterion (fewer number of dimensions), (b) the high correlation between the Paranoid and Positive factors in the four-factor model of Swiss sample, and (c) the lower weight of the factor loadings and no statistical significance of four items in the four-factor model, the Raine at al.'s [37] three-factor model was selected as the most adequate. Table 1 shows the standardized factor loadings in both samples for this hypothetical model.

**Table 2:** Goodness-of-fit indices for the theoretical models proposed.

Models	?^2^	*df*	CFI	TLI	RMSEA	WRMR
*Spain*						
One-factor	574.81	209	0.87	0.86	0.08	1.30
Siever and Gunderson, [65], Two-factor	531.26	208	0.89	0.875	0.08	1.26
Raine et al., [37], Three-factor	405.77	202	0.93	0.92	0.06	1.08
Raine et al., [28], Three-factor	466.66	206	0.91	0.90	0.07	1.18
Stefanis et al., [38], Four-factor (paranoid)	401.99	199	0.93	0.92	0.06	1.08
*Switzerland*						
One-factor	383.02	209	0.91	0.93	0.06	1.05
Siever and Gunderson, [65], Two-factor	337.25	208	0.95	0.95	0.05	0.99
Raine et al., [37], Three-factor	244.32	202	0.98	0.98	0.03	0.83
Raine et al., [28], Three-factor	283.30	206	0.97	0.97	0.04	0.91
Stefanis et al., [38], Four-factor (paranoid)	242.64	199	0.98	0.98	0.03	0.83

χ^2^ = Chi square; *df*  =  degrees of freedom; CFI  =  Comparative Fit Index; TLI  =  Tucker-Lewis Index; RMSEA  =  Root Mean Square Error of Approximation; WRMR =  Weighted Root Mean Square Residual. Note:

### Measurement invariance of the SPQ-B scores across the two samples

Measurement invariance across Spanish and Swiss adolescents was studied for the model hypothesized by Raine et al., [37]. The configural invariance model, in which no equality constraints were imposed, showed an adequate fit to the data (see table 3). Next, a strong invariance model was tested with the item thresholds and factor loadings being constrained to equality across groups. The ΔCFI between the constrained and the unconstrained models was over 0.01, indicating that strong invariance was not supported. The modification indices suggested that the thresholds of five items (2, 8, 15, 17, and 19) constituted the largest source of misfit, and that these thresholds should be relaxed. This partial strong invariance model showed adequate fit to the data. In this case, the ΔCFI was equal to 0.01, so that, according to the recommendations by Cheung and Rensvold [60], partial strong invariance was accepted. Hence, the results support configural, and partial strong invariance of the SPQ-B scores across the two samples from different countries.

**Table 3:** Measurement invariance across groups for three-dimensional model proposed by Raine et al., [37].

	?^2^	*df*	CFI	TLI	RMSEA	WRMR	ΔCFI
*Groups*							
Spain (*n* = 261)	405.77	202	0.93	0.92	0.06	1.08	
Switzerland (*n* = 241)	244.32	202	0.98	0.98	0.03	0.83	
*Measurement invariance*							
1. Configural invariance	254.44	154	0.93	0.95	0.05	1.38	
2. Strong factorial invariance	285.56	156	0.90	0.92	0.06	1.67	+0.01
2a. Partial strong factorial invariance: freeing intercepts (2,8,15,17,19)	274.62	154	0.92	0.94	0.05	1.51	−0.01

χ^2^ = Chi square; *df*  =  degrees of freedom; CFI  =  Comparative Fit Index; TLI  =  Tucker-Lewis Index; RMSEA  =  Root Mean Square Error of Approximation; WRMR =  Weighted Root Mean Square Residual Note:

### Comparisons in the latent means

Latent mean differences across groups were estimated, fixing the latent mean values to zero in the Spanish sample. For comparisons among groups in the latent means, statistical significance was based on the *z* statistic. The group in which the latent mean was fixed to zero was considered as the reference group. The comparison across groups in latent means revealed statistically significant differences in the Interpersonal dimension of SPQ-B. Thus, the comparison across groups in latent means indicated that, on average, Swiss teenagers scored 0.357 units below the Spanish in the Interpersonal dimension (−0.357; *p*≤0.05). For Cognitive-Perceptual and Disorganization dimensions statistically significant differences were not found.

## Discussion and Conclusion

The main goal of the present research was to analyze the measurement invariance of the Schizotypal Personality Questionnaire-Brief (SPQ-B) [28] scores across Spanish and Swiss adolescents. The results support configural and partial strong measurement invariance of the SPQ-B scores across the two samples, and provide preliminary validity for the factorial equivalence of schizotypy across countries. These results are of essential importance, not only for the study of the construct validity of schizotypy and subclinical psychosis phenotype, but also for the application and utility of the SPQ-B in cross-cultural research and our understanding of the phenotypic expression of schizotypy from a developmental perspective.

The results of the study indicate that the structure underlying the schizotypal personality in adolescents fits both Raine et al.'s [37] three-factor model and Stefanis et al.'s [38] four-factor model reasonably well, and that there are considerable parallels between them. However, for this study, due to the parsimony criterion, the high correlation between the Paranoid and Positive factors in the four-factor model, and the lower weights of the standardized factor loadings in the Stefanis et al.'s model, Raine et al.'s model [37] was chosen as the most adequate. Previous studies using the SPQ-B have found similar results [28]-[30], [32], [34]–[36]. For instance, Fonseca-Pedrero et al., [34] conducted a factorial study of the SPQ-B in a large sample of adolescents and young adults, finding that Raine et al.'s [37] model yielded the best goodness-of-fit indices in comparison to other models hypothesized. Likewise, these results are convergent with those found using the SPQ [39]–[45]. In addition, this model is consistent with the structure of symptoms found in patients with schizophrenia [63], revealing phenotypic parallels between clinical and non-clinical populations.

Second, the hypothesized dimensional model proposed by Raine et al., [37] was equivalent across the two samples. It is noteworthy that, although the goodness-of-fit indices for the partial strong invariance model were adequate, several item thresholds were relaxed, suggesting a possible bias of measurement (e.g., differential item functioning). Previous studies using the Wisconsin Schizotypy Scales (WSS) found that the schizotypy dimensions were invariant across cultures [2]. For instance, Kwapil et al. [64], using the WSS in Spanish and American samples, found that the hypothesized two factor model (Positive and Negative) was invariant across groups. These preliminary data appear to support the cross-cultural validity of two different schizotypy measurement instruments (WSS and SPQ-B). In addition, in the present study, Spanish participants scored higher on Interpersonal dimension than Swiss when latent means were compared. These results are of crucial relevance when it comes to setting cut-off points for the purpose of detecting participants at risk of psychosis in different countries. In this regard, the results appear to underline the importance of culture when setting cut-off points, with the SPQ-B, at least for what concerns its Interpersonal dimension. Furthermore, this result reflects that the construct measured has, at least, the same structure and meaning across the groups compared.

It should be stressed that if measurement invariance does not hold, the suggestion is that the validity of such scores as measures of schizotypy should be questioned. As such, it is critical for measurement invariance conclusions to be based on statistically sound results. The comparability between different groups only makes sense if it can be guaranteed that participants interpret and understand the latent construct in a similar manner. Hence, from a psychometric point of view, the study of measurement invariance is a prerequisite for performing any group comparisons [46], [47]. When the data supporting the dimensional structure underlying the SPQ-B scores is invariant across the groups, we are asserting that participants interpret and respond to the items in the measurement instrument in a similar manner. We are also asserting that the factorial structure found is equivalent and presented in the same metric across groups. Therefore, if any difference in the latent mean score is found, we can be sure that such difference is a result of a true difference in the latent variable, and not a measurement artefact. Previous studies in schizotypy research have not explored the possible existence of differences between the latent means of the schizotypy dimensions across countries [64]. Based on these findings, future research should be further pursued.

The results of the present study should be interpreted in the light of the following limitations. First, the SPQ-B is a brief measurement instrument for the assessment of schizotypy in which multiple factorial models cannot be tested. Second, we did not use a response infrequency scale for eliminating data from participants who may have responded dishonestly or randomly to the self-report items. Third, no information was gathered regarding the participants' psychiatric morbidity or the use or abuse of substances, aspects that may partially influence the results. Finally, the present study used country as a proxy for culture. Further studies investigating cultural differences would benefit from including measures of cultural values and beliefs in their assessments. Results found in the present study have clear implications for the research on the construct validity of schizotypy across countries. Future research should continue to advance in the study of measurement invariance of schizotypal dimensions across other cultures (i.e., non-Western), as well as exploring other measurement instruments (i.e., CAPE-42, PDI-21), in order to guaranteeing the comparability and cross-cultural equivalence of this construct.
